# Trigger Points and Contracture/Contraction Knots: What’s in a Name? Reply to Dommerholt, J.; Gerwin, R.D. Contracture Knots vs. Trigger Points. Comment on “Ball et al. Ultrasound Confirmation of the Multiple Loci Hypothesis of the Myofascial Trigger Point and the Diagnostic Importance of Specificity in the Elicitation of the Local Twitch Response. *Diagnostics* 2022, *12*, 321”

**DOI:** 10.3390/diagnostics12102366

**Published:** 2022-09-29

**Authors:** Andrew Ball, Thomas Perreault, César Fernández-de-las-Peñas, Michael Agnone, Jordan Spennato

**Affiliations:** 1 Atrium Health, Carolinas Rehabilitation, Charlotte, NC 28211, USA; 2 Myopain Seminars, 4405 East-West Highway, Suite 401, Bethesda, MD 20814, USA; 3 NxtGen Institute, 2138 Scenic Highway, Snellville, GA 30078, USA; 4 Wentworth-Douglass Hospital Rehab Services at Dover, 789 Central Avenue, Dover, NH 03820, USA; 5 Department of Physical Therapy, Occupational Therapy, Rehabilitation and Physical Medicine, Universidad Rey Juan Carlos, 28922 Madrid, Spain

**Keywords:** trigger point, contracture knot, contraction knot, ultrasound

## Abstract

We are responding to the comment by Dommerholt and Gerwin that we have reverse-defined “myofascial trigger point” (MTrP) and “contracture/contraction knot.” In attempting to maintain philosophical agreement with specific and implied aspects of their integrated hypothesis of trigger-point formation (namely a MTrP being ischemic and hypoxic), we referred to the MTrP as the small hyperechoic signal rather than the larger hypoechoic (and therefore hyperperfused) structure surrounding it. It was never our intent to re-define nor contribute to confusion. In making this concession with respect to Dommerholt and Gerwin’s preferred nomenclature, however, we must instead now reconcile what we image as a hypoechoic (and therefore hyperperfused) MTrP with it being concurrently hypoxic.

## 1. Introduction

We welcome the comment by Dommerholt and Gerwin [1]. Based upon their comment, there are clearly subtle differences in interpretation of the literature. We believe it to be most fruitful to the reader to address the primary concern of Dommerholt and Gerwin—that we introduced the reverse-defining of trigger points versus the contracture knot. To this point, we will illustrate that we were not the first to do so, but we merely elucidated this confusion. Although we described trigger points as occurring at motor endplates in an effort to maintain the idea that the trigger point is hypoxic and ischemic, it was not our intent to reverse-define, nor do we have particular disagreement with their concern on that matter. For the sake of consistency and clarity, we will use their nomenclature throughout the current response. In doing so, we are also forced to point out that the palpable trigger point is consistently imaged as hypoechoic with contemporary imaging systems, and therefore theoretically *hyper*perfused (although smaller hyperechoic speckles may exist *within* it). In short, if Dommerholt and Gerwin insist on a particular nomenclature, they then invite the fundamental challenge to their own integrated hypothesis of trigger-point formation [2] and (albeit indirectly) ideas about how trigger-point dry needling may exert some of its local therapeutic effects that we struggled to circumvent. It was our desire to avoid what may be misinterpreted as a direct challenge to their work and instead resolve what we feel to be a relatively minor issue through future collaborative endeavors. Even now, we are reluctant and uncomfortable to spotlight the above as we view their hypothesis as the most comprehensive and evidence-informed available to date. We wish to refine and elevate their work, and not be viewed as standing in opposition to it. That being said, there are no accidental ultrasound echoes. We must reconcile the ultrasound evidence suggesting that trigger points are hypoechoic and therefore hyperperfused with the evidence suggesting that trigger points are hypoxic. The two ideas would seem in mutually exclusive opposition. Anisotropy (e.g., image-error) is certainly a possibility, but an increasingly unlikely explanation given the quality of contemporary ultrasound systems versus those from even a few years ago.

## 2. Nomenclature and Ultrasound Imaging

### 2.1. The Hypoechoic Myofascial Trigger Point

Dommerholt and Gerwin argue from the perspective that the terms trigger point and contracture/contraction knot have not been used interchangeably and that no confusion exists in the literature nor in clinical practice. We respectfully disagree. Although most studies refer to the myofascial trigger point (MTrP) as the larger palpable entity that appears hypoechoic upon ultrasound imaging [3], others refer to the MTrP as alternatively hyperechoic [4]. We were initially unable to locate a copy of Dr. Gerwin’s 1997 article entitled “Ultrasound identification of the myofascial trigger point” [5], but we were able to review the interpretation of others. As Thomas and Shankar [4] reference Gerwin [5] in describing a MTrP as hyperechoic, it was initially unclear if Dr. Gerwin described a contracture/contraction knot rather than a MTrP, or if (like Thomas and Shankar), with inferior ultrasound technology (and in contrast and conflict to other researchers) [3], he had identified MTrPs as hyperechoic. Lack of consensus regarding MTrP appearing bright (hyperechoic) or dark (hypoechoic) is a source of confusion in that the reader can never be quite certain if the author is referencing the trigger point, referencing the contraction/contracture knot, or has made an error in interpretation of the image.

### 2.2. The Hyperechoic Contracture/Contraction Knot

Further adding to the nomenclature confusion that Dommerholt and Gerwin claim not to exist, some authors have in fact used the term MTrP to describe something much smaller occurring at the motor endplate. For example, we would argue that what was visualized in Liu’s [6] study was most probably a collection of contracture/contraction knots versus what they (mis)labeled a MTrP. For our part, we simply utilized Liu’s nomenclature because it more closely aligned with Gerwin et al.’s hypothesis that a MTrP should be both hypoperfused and hypoxic [2]. The charge that *we* reversed definitions of the terms trigger point and contracture/contraction knot for no apparent reason—rather than once again illuminating confusion—is therefore both historically and factually inaccurate. It is further confusing given that we did so in a specific attempt to preserve Gerwin and Dommerholt’s integrated hypothesis of trigger-point formation [2], which we the authors support (a potentially problematic idea if the MTrP is visualized under ultrasound as hypoechoic/hyperperfused).This particular example of so-called “definition reversal” has existed in the literature for several years without (to our knowledge) challenge from Dommerholt, Gerwin, or anyone else.

We were delighted to ultimately locate a copy of Gerwin’s article entitled “Ultrasound identification of the myofascial trigger point,” expecting for the hyperechoic signal described to be anisotropy as it would have been unlikely for a study published in 1997 to have used technology capable of imaging a small hyperechoic contracture knot within the MTrP. Gerwin [5] not only did not identify a hyperechoic trigger point as others have erroneously referenced [4], but despite the title of the article, did not image a MTrP at all. What he identified in his image was (in keeping with some of our images) a hypoechoic taught band [5]. In a more recent study, Shankar and Reddy [7] used two and three dimensional ultrasound imaging to identify not hypoechoic, but hyperechoic taut bands. We find it somewhat dubious that we are charged with contributing to the confusion regarding nomenclature—rather than having simply clarified it.

## 3. Ultrasound Echo and Interpretations of Ventilation and Perfusion

**Figure 1:**
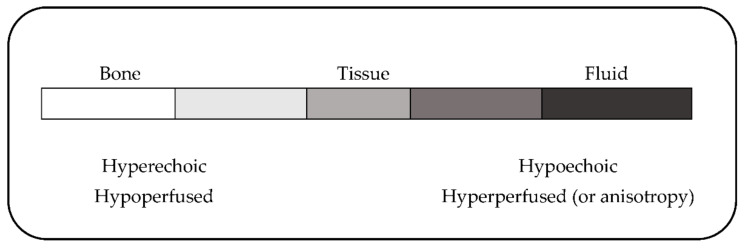
Illustration of musculoskeletal ultrasound interpretation. Note that fluid is always black andtissue is always grey. Bone, being the densest tissue, and least perfused will appear the brightest grey/white. Trigger points are consistently imaged as hypoechoic and therefore, barring anisotropy/image error, must be hyperferfused.

We appreciate the comment by Dommerholt and Gerwin regarding the interpretation of hyperperfusion as being directly related to oxygenation. We agree that we took some liberties in that regard. Dommerholt and Gerwin are quite correct that Shah et al. measured chemical mediators within the trigger point and not perfusion per se [8], but the published image from Sikdar labels the trigger point as hypoechoic [3]. As illustrated in Figure 1, hypoechoic structures are either fluid/perfusion or anisotropy (aka image-error) [9,10,11,12,13]. However, oxygenation and perfusion are generally considered to be directly related in healthy lean muscle [14], with hyperperfusion generally interpreted as hyperoxygenation. This may not universally be the case—particularly where hypercontracted muscle and abnormal partial pressures are involved [15]. According to Henry’s Law, the relationship between ventilation and perfusion, specifically in muscle, is far more complex [16,17,18]. Beyond that potential over-generalization, it is difficult to take their critique constructively based upon their incorrect use of supporting references.

In the study cited by Brückle et al. [19], polarographic needle probes were used to study oxygen flux at the tissue level, likely to assess ischemia and/or hypoxia within the muscles of study subjects. However, blood perfusion was not directly measured. Further interpretation of this reference was not possible for us due to the fact that the only available full text we could locate was in the German language, which none of the authors can interpret. Of note from the translated abstract is the fact that Brückle uses the term “myogelosis,” a term reportedly meant to infer the presence of nodules that are high viscosity muscle colloids. It would be informative to inquire of Dommerholt and Gerwin what their thoughts may be with regard to the potentially confusing nomenclature of the term myogelosis when the apparent structure studied is now more commonly labeled a MTrP.

A similar study by Strobel et al. also used polarographic oxygen fine-needle probes to assess tissue oxygen tension of a myogelosis in three patients with myofascial pain syndrome and trigger points. They reported tissue oxygen tension was increased at the border of the myogelosis but steadily decreased towards its center with tissue p02 values indicating hypoxia [20]. Exactly like the study by Brückle cited by Dommerholt and Gerwin, however, blood perfusion of the TrP was not directly measured. We do understand that this could imply “hyperperfusion outside and hypoperfusion inside”, but again perfusion was not measured. We feel it is necessary to expose an error in the response of Dommerholt and Gerwin’ when they claimed “unlike Brückle et al., who, in 1990, observed hyperperfusion outside TrPs and hypoperfusion within TrPs.” Furthermore, it is not entirely clear if these researchers were truly measuring what imaging would suggest to be the hypoechoic and theoretically hyperperfused MTrP, or the hyperechoic contracture/contraction knot within.

## 4. Speckles: Are They Contracture Knots?

To Dommerholt and Gerwin’s point, it is certainly possible that the speckles we observed were simply fascia and collagen with no relation to trigger points, but these speckles were somewhat smaller and more hyperechoic than what was observed in the surrounding musculature. Alternatively, it is an exciting prospect to consider that they *could* be. We cannot say with certainty that the hyperechoic speckles are contracture/contraction knots. We can, however, say that there seem to be small, hyperechoic structures within the hypoechoic and hyperperfused trigger point. We believe that the limitations section sufficiently addressed the possibility that these speckles may have no relation to contracture/contraction knots. What we did not note in the paper is that the speckles appear to be a bit more hyperechoic within the hypoechoic area of hypoperfusion than within the “normal” muscle. That aspect of the images does not show up well in print, and furthermore we cannot be convinced that it’s not something of an optical illusion against the darker muscular environment within the trigger point.

## 5. Implications for Future Research

Taken inclusively, we believe this to result in a body of literature that could be re-assessed with exciting possibility for research and microphysiologic understanding of the trigger point environment regardless of where the nomenclature ultimately lands. With respect to microdialysis studies for example, where *exactly* was the tip of the needle placed [8,19,20]? It is unclear if microdialysis studies describe differences between the contracture/contraction knot and the surrounding MTrP environment, or the MTrP and the muscular environment surrounding the trigger point. Was the tip of the needle in the MTrP, or more specifically in the smaller contracture/contraction knot? Given that we have imaged the MTrP to be hypoechoic with smaller contracture/contraction knot speckles within, it would stand to reason that the chemical mediators, oxygenation, and/or perfusion of contracture/contraction knots versus trigger points are similarly profoundly different. In other words, a needle placed merely in a trigger point versus a needle placed more specifically into a contracture/contraction knot may yield drastically different research outcomes and/or clinical outcomes.

We find Sikdar and Shah’s hypothesis that with respect to a MTrP, “The hypoechoic and stiffer nodules may be indicative of contraction knots resulting from increased muscle fiber contraction and recruitment, local injury, and/or localized regions of ischemia” unsatisfactory [21] because a hypoechoic ultrasound signature suggests hyperperfusion, not ischemia. We propose that either that Shah’s needle tip was more specifically in a contracture/contraction knot (hyperechoic and hypoperfused) versus the larger MTrP, or alternatively that (to account for hypoechoic MTrP that is *also* hypoxic) a slight refinement of our understanding of trigger-point microphysiology is required. The questioning and resolution may not have immediate clinical relevance, nor result in the need for any significant modification of the integrated hypothesis of trigger point formation [2], but surely has relevance seeking to understand mechanisms of how clinical modalities such as trigger point injection, dry needling, and intramuscular electrical stimulation exert their therapeutic effect.

## 6. Conclusions

In conclusion, we would like to stress that a small case report with a handful of patients and images should never be mis-interpreted by the reader as a definitive work and conclusion of a professional conversation, but rather the grounded theory hypothesis-generating beginning of it. In that respect, we genuinely appreciate the comment by Drs. Dommerholt and Gerwin and stand by the work in serving its purpose in that regard.
